# The efficacy and safety of dalpiciclib, a cyclin-dependent kinase 4/6 inhibitor, in patients with advanced head and neck mucosal melanoma harboring CDK4 amplification

**DOI:** 10.1186/s12916-024-03431-x

**Published:** 2024-05-29

**Authors:** Chaoji Shi, Houyu Ju, Rong Zhou, Shengming Xu, Yunteng Wu, Ziyue Gu, Ying Wang, Wanling Chen, Xinyi Huang, Yong Han, Shuyang Sun, Chuwen Li, Min Wang, Guoyu Zhou, Zhiyuan Zhang, Jiang Li, Guoxin Ren

**Affiliations:** 1 Department of Oral and Maxillofacial-Head Neck Oncology, College of Stomatology, Shanghai Ninth People’s Hospital, Shanghai Jiao Tong University School of Medicine, Number 639, Zhi-Zao-Ju Road, Shanghai, 200011 People’s Republic of China; 2 National Clinical Research Center for Oral Diseases, National Center for Stomatology, Shanghai, 200011 People’s Republic of China; 3 Shanghai Key Laboratory of Stomatology &, Shanghai Research Institute of Stomatology, Shanghai, 200011 People’s Republic of China; 4 Research Unit of Oral and Maxillofacial Regenerative Medicine, Chinese Academy of Medical Sciences, Shanghai, 200011 People’s Republic of China; 5 Clinical Research & Development, Jiangsu Hengrui Pharmaceuticals Co., Ltd, Shanghai, 200011 People’s Republic of China; 6 Department of Oral Pathology, School of Medicine, Ninth People’s Hospital, Shanghai Jiao Tong University, Number 639, Zhi-Zao-Ju Road, Shanghai, 200011 People’s Republic of China

**Keywords:** CDK4/6 inhibitor, Dalpiciclib, Head and neck mucosal melanoma, Clinical trial, CDK4 amplification

## Abstract

**Background:**

Mucosal melanoma (MM) is a rare but devastating subtype of melanoma. Our previous studies have demonstrated robust anti-tumor effects of cyclin-dependent kinase 4/6 (CDK 4/6) inhibitors in head and neck MM (HNMM) patient-derived xenograft models with CDK4 amplification. Herein, we aimed to investigate the efficacy and safety of dalpiciclib (SHR6390), a CDK4/6 inhibitor, in HNMM patients harboring CDK4 amplification.

**Methods:**

The anti-tumor efficacy of dalpiciclib was assessed by HNMM patient-derived xenograft (PDX) models and patient-derived tumor cells (PDC) in vivo and in vitro. Immunohistochemical analyses and western blot were then performed to assess the markers of cell proliferation and CDK4/6 signaling pathway. For the clinical trial, advanced recurrent and/or metastatic HNMM patients with CDK4 amplification were treated with dalpiciclib 125 mg once daily for 21 consecutive days in 28-day cycles. The primary endpoint was disease control rate (DCR). Secondary endpoints included safety, objective response rate (ORR), progression-free survival (PFS), and overall survival (OS).

**Results:**

Dalpiciclib profoundly suppressed growth of HNMM-PDX and PDC with CDK4 amplification, whereas it showed relatively weak suppression in those with CDK4 wild type compared with vehicle. And dalpiciclib resulted in a remarkable reduction in the expression levels of Ki-67 and phosphorylated Rb compared with control group. In the clinical trial, a total of 17 patients were enrolled, and 16 patients were evaluable. The ORR was 6.3%, and the DCR was 81.3%. The estimated median PFS was 9.9 months (95% CI, 4.8-NA), and the median OS was not reached. The rate of OS at 12 months and 24 months was 68.8% (95% CI, 0.494–0.957) and 51.6% (95% CI, 0.307–0.866), respectively. The most frequent adverse events were neutrophil count decrease, white blood cell count decrease, and fatigue.

**Conclusions:**

Dalpiciclib was well-tolerated and displayed a durable benefit for HNMM patients with CDK4 amplification in this study. Further studies on CDK4 inhibitors and its combination strategy for MM are worth further exploration.

**Trial registration:**

ChiCTR2000031608.

**Supplementary Information:**

The online version contains supplementary material available at 10.1186/s12916-024-03431-x.

## Background

Mucosal melanoma (MM) is a rare and devastating subtype of melanoma, which is common in the Asian population [1]. The nasal cavity and paranasal sinuses, oral cavity, and oropharynx and any other part of the mucosal surface lining are the primary sites for mucosal melanoma [2, 3]. The 5-year survival rate of MM is less than 25%, much lower than that of other melanoma subtypes, possibly because of its high rate of recurrence and metastasis, as well as a lack of effective therapies [4–6]. Although targeted agents like BRAF and KIT inhibitors and PD-1/PD-L1 immune checkpoint inhibitor (ICI) have revolutionized clinical management of melanoma, these treatments are much less effective in MM [7]. The efficacy outcome of ICI was much poorer in MM, with a response rate of 19–30% to anti-PD-1 monotherapy, compared with a 40% response rate in cutaneous melanoma [8, 9]. Considering the serious therapeutic dilemma, there is an unmet need for developing more effective treatment strategies for MM [10].

Several studies have indicated that inhibition of CDK4 pathway activity may be a potential therapeutic option for melanoma, including MM. Our previous whole genome sequencing (WGS) study of 65 MMs originated from the head and neck region has revealed that the dysregulation of cell cycle progression is a key genetic feature in MM. Over 50% of the HNMM patients harbored CDK4 amplification, which were demonstrated in an independent validation cohort of 80 HNMM samples by using droplet digital PCR and other study [3, 11]. However, the ratio of CDK4 amplification in cutaneous melanoma was only 4.3% (7/140) according to Hayward et al. reports [12].

Additionally, our preclinical results have demonstrated robust anti-tumor effects of CDK 4/6 inhibitor in HNMM patient-derived xenograft models with CDK4 amplification [3]. Given that a subset of MMs harbor CDK4 amplifications, these patients may be promising candidates for CDK4-targeted treatments [13]. However, to our knowledge, no clinical trial has focused on the CDK4/6 inhibitors in patients with MM so far.

Dalpiciclib (SHR6390) is a new, orally administered, selective CDK4/6 inhibitor, which has been approved by the National Medical Products Administration for the treatment of hormone receptor-positive, human epidermal growth factor receptor 2 (HER2)-negative advanced breast cancer [14, 15]. Dalpiciclib also exerted potential antitumor activity in other malignancies, such as esophageal squamous cell carcinoma and other RB-positive cancer cell lines [16, 17]. To test the hypothesis that CDK4 inhibitors could target MM with CDK4 amplification, we first assessed the anti-tumor activity of dalpiciclib via HNMM PDX and PDC models. Based on the preclinical results, we evaluated the efficacy and safety of the single-agent, dalpiciclib, in HNMM patients with CDK4 amplification in an investigator-initiated, molecularly stratified phase 2 trial.

## Methods

### In vivo and in vitro evaluation of therapeutic agents

PDC, PDX, and in vivo drug sensitivity experiments referenced our previous reports [3, 18, 19]. Fresh tissue biopsy of the donor was subcutaneously inoculated into 5-week-old nude mice (BALB/c nude).

Xenograft from established PDX model was trimmed into about 20-mm^3^ fragments for subcutaneous implantation in nude mice. Xenograft-bearing mice with an average tumor volume of 100 to 150 mm^3^ were randomized into the control arm and the treatment arm on the basis of tumor size, tumor growth rate, and mouse body weight and were treated with vehicle (the drug solvent), palbociclib (orally, daily), and dalpiciclib (orally, daily) at a dose of 100 mg/kg. Tumor size (*V* = length × width^2^/2) and body weight measurements for mice were taken twice weekly respectively. Tumor growth inhibition (TGI) was defined as the following formula: % growth inhibition = 100 × [1 − (*V*_t treated_ − *V*_t0 treated_)/(*V*_t vehicle_ − *V*_t0 vehicle_)], where *V*_t_ represents the average tumor volume at the end of study, and *V*_t0_ represents the average tumor volume on the first day of treatment. Animal care and experiments were performed under the approval and supervision of the Institutional Animal Care and Use Committee (IACUC) of the Shanghai Jiao Tong University School of Medicine.

PDC cultures were performed as described [20]. The dissected primary tumor or tumor from PDX was washed 3 times in precooling PBS containing 2% penicillin/streptomycin, 1% gentamicin, and 1% amphotericin B. Tumor tissues were minced in a 6-cm dish and were centrifuged at 4 °C for 3 min at 200 g. The supernatant was aspirated and digested to a single-cell suspension using a digestion solution (collagenase IV (1 mg/mL), hyaluronidase (200 U/L), DNase I (200 U/L), and 0.05% Tyrpsin-EDTA in RPMI 1640 medium) and were processed with a gentle MACS Dissociator (Miltenyi Biotec) about 30 min according to the manufacturer’s instructions. After the digestion, a culture medium containing serum was added to terminate the digestion of the tissue. The cells were filtered through 30-nm cell strainers and resuspended in PBS supplemented with 2% FBS. The digested tissue was sequentially filtered through 100-μm and 70-μm filter mesh and washed with tissue culture medium. Cells were washed twice in sterile PBS and counted in a hematocytometer and then resuspended in a pre-warmed medium RPMI-1640 medium with 20% fetal bovine serum (FBS, Gibco) at 10^4^ cells/ml and 1% PS and inoculated in 10-cm dishes.

In this study, fresh tissue biopsies for PDX and PDC experiments were not obtained from some of the enrolled patients in the phase II trial.

### Western blotting

Western blotting was performed as our previous study [3]. The brief description is as follows. Freshly frozen xenograft tissue and PDC cells were lysed using 1% SDS solution (Beyotime), containing 1% phosphatase and proteinase inhibitor cocktail (Bimake). Approximately 200 mg of xenograft tissue was homogenized and lysed using 0.5 mL of lysis buffer. The protein concentration of individual samples was determined with BCA Protein Assay Kit (Beyotime). An equal quantity of protein was separated by 10% SDS-PAGE and transferred onto a transfer membrane (Millipore) and was blocked with 5% nonfat dry milk for 1 h at room temperature. After blocking with non-fat milk, the membranes were incubated with primary antibodies overnight at 4 °C: Rb (#ab24, Abcam), GAPDH (#2118, CST), phospho-Rb (S807/811, #8516, CST), CDK4 (#108,357, Abcam), CDK4 (#124,821, Abcam), and Cyclin D1(#55,506, CST). Horseradish peroxidase and secondary antibodies were used at room temperature for 1 h the following day. The chemiluminescence signal was developed with ECL-plus reagent (Beyotime) and detected by autoradiography.

### Immunohistochemistry

Immunohistochemistry (IHC) was performed referenced our previous reports [16]. The brief description is as follows: 4 μm tumor sections cut from FFPE tissue blocks using monoclonal antibodies Ki-67 (MX006, Fuzhou Maixin Biotech) and phospho-Rb (S807/811, #8516, CST) on an automated immunostainer (AutostainerLink 48, Agilent Technologies, California). The staining result was then evaluated independently by two pathologists. Any discrepant cases were reevaluated by consensus review.

### Study design

This was a single-arm open-label study of dalpiciclib in patients with recurrent and/or metastatic MM of head and neck harboring CDK4 amplification. The study was registered in the Chinese Clinical Trials Registry Platform (ChiCTR2000031608) and approved by the Ethics Committee of Shanghai Ninth People’s Hospital, Shanghai Jiao Tong University School of Medicine (SH9H-2019-T282-6). The primary endpoint was the disease control rate (DCR) lasting at least 8 weeks after initiation of treatment, defined as confirmed complete response (CR) or partial response (PR), or stable disease (SD) for ≥ 8 weeks according to RECIST v1.1, as a proportion of the total number of patients who received at least one cycle of the study drug. The secondary endpoints were adverse events (AEs), objective response rate (ORR), progression-free survival (PFS), and overall survival (OS).

### Patient eligibility

Eligible patients had a diagnosis of recurrent and/or metastatic unresectable mucosal melanoma of head and neck with CDK4 amplification. Unresectable patients must have failed at least one systemic treatment, including having received surgery and adjuvant treatment with curative intent and failure of adjuvant therapy. All patients had evidence of clinical disease progression before enrolling onto this trial. Other eligibility criteria included age > 18 years, Eastern Oncology Cooperative Group (ECOG) performance status of 0 or 1, and adequate organ function, at least one measurable target lesion following Response Evaluation Criteria in Solid Tumors (RECIST v1.1) guidelines. The key exclusion criteria were prior or current treatment with any kind of CDK4/6 inhibitors, cytotoxic chemotherapy within 4 weeks or any other anti-tumor therapy within 4 weeks, untreated or unstable brain metastases (as judged by the investigator), and gastrointestinal conditions that could affect drug absorption.

### Treatment

Patients with advanced MM, harboring CDK4 gene amplification, were given dalpiciclib orally (125 mg QD) for 3 weeks, followed by 1 week break in each 4-week cycle according to the usage in dalpiciclib labeling for breast cancer. The 4-week treatment cycle was continued until intolerability, disease progression, or withdrawal of consent. Up to 2 dose reductions (each 25 mg decrements) for dalpiciclib were allowed, and dose delays for AEs were permitted. Patients who had disease control beyond 1 year could continue to receive dalpiciclib off-trial until disease progression, unacceptable toxicity, or withdrawal of consent.

### Response and safety assessments

Response was assessed by radiographic examinations every 6 to 8 weeks. Radiographic examinations were assessed by the investigator and diagnostic imaging specialists according to RECIST version 1.1. Safety was assessed by the incidence of AEs, reported according to the Common Terminology Criteria for Adverse Events (CTCAE), version 5.0, including physical examination, routine laboratory tests, and toxicity assessments at the end of each cycle.

### CDK4 amplification assessment

CDK4 amplification testing by fluorescence in situ hybridization (FISH) was performed on FFPE tissue sections using commercial probes, ZytoLight SPEC CDK4/CEN12 Dual Color Probe (Zytovision, Bremerhaven, Germany), according to our previous research [3]. The experimental procedures were performed according to the manufacturer’s instructions. Amplification was defined by examining the CDK4/CEN12 ratio followed by the average CDK4 copy number: CDK4/CEN12 ratio < 2.5 with an average CDK4 copy number ≥ 5.0 signals per nucleus or a ratio > 2.5 or an uncountable sample due to clustering of green signals.

### DNA extraction and whole-exome sequencing

Genomic DNA was extracted from formalin-fixed paraffin embedded (FFPE) samples Maxwell 16 FFPE Plus LEV DNA Purification Kit (Promega) according to the manufacturer’s instructions in Sequanta Technologies at Shanghai. Whole-exome capture libraries were constructed from extracted tumor DNA. Then, the integrity and concentration of the total DNA were determined by agarose electrophoresis and Qubit 3.0 fluorometer dsDNA HS Assay (Thermo Fisher Scientific). OD260/OD280 was measured by NanoDrop2000 (Thermo Fisher Scientific). About 300 ng high-quality DNA sample (OD260/280 = 1.8 ~ 2.0) was used to construct sequencing library. DNA concentration of the enriched sequencing libraries was measured with the Qubit 2.0 fluorometer dsDNA HS Assay (Thermo Fisher Scientific). Size distribution of the resulting sequencing libraries was analyzed using Agilent BioAnalyzer 2100 (Agilent). Sequencing was then performed with paired-end 2 × 150 base reads on the Illumina NovaSeq6000 platform in Sequanta Technologies Co., Ltd. Raw FASTQ files were first processed by a proprietary algorithm to filter out mouse sequence contaminations. Somatic SNVs and InDels were detected with Sentieon TNseq. Mutations in low-complexity regions, such as tandem repeat and highly homologous regions, were filtered out. Low-confidence variants were removed if any one of the following criteria was not satisfied: total depth > 10, alternative allele depth > 3, and mutation frequency > 0.01. All high-confidence mutations were then annotated with ANNOVA (Version 2016–02-01). A CNV kit was used to analyze somatic copy number variations (CNVs). CNVs were called with a CNV kit by comparing the normalized tumor and normal data. Regions with an absolute log2 copy number ratio of at least 0.58 (= log2(1.5)) were viewed as losses or gains. Tumor mutation burden (TMB) was calculated with the total numbers of non-synonymous SNVs per megabase of coding regions.

### Statistical analysis

A sample size is calculated with the assumption of a baseline DCR of 10% of advanced recurrent metastatic melanoma, and the target treatment effect on DCR is 40%; a sample size of 14 will provide approximately 90% power with a one-sided alpha of 0.05. Assuming a dropout rate of 15%, an initial 17-patient enrollment was planned. Summary data are reported for in vivo and in vitro data, patient baseline demographics and efficacy and safety data. Data are presented descriptively, with categorical data presented as *N* (%), and continuous data are presented as the median and range, unless differently indicated. The Shapiro Wilk (S-W) test was used for testing the normality distribution of data. Student’s *t* test was performed for in vivo and in vitro evaluation. A *P* value < 0.05 was considered significant. The median PFS and OS, 12-month survival rate, 24-month survival rate, and their 95% confidence intervals (95% CI) are estimated using the Kaplan–Meier method. The sample size calculation and statistical analysis were performed using the PASS (version 15.0) and SPSS (version 25.0). All patients who received at least 1 cycle of treatment (28 days) of the study drug were included in full analysis set (FAS), and patients who received at least 1 dose of the study treatment were included in the safety analysis set (SAS). Efficacy were analyzed in FAS, while safety was analyzed in SAS.

## Results

### Evaluation of dalpiciclib in HNMM-PDX/PDC models

**Fig. 1:**
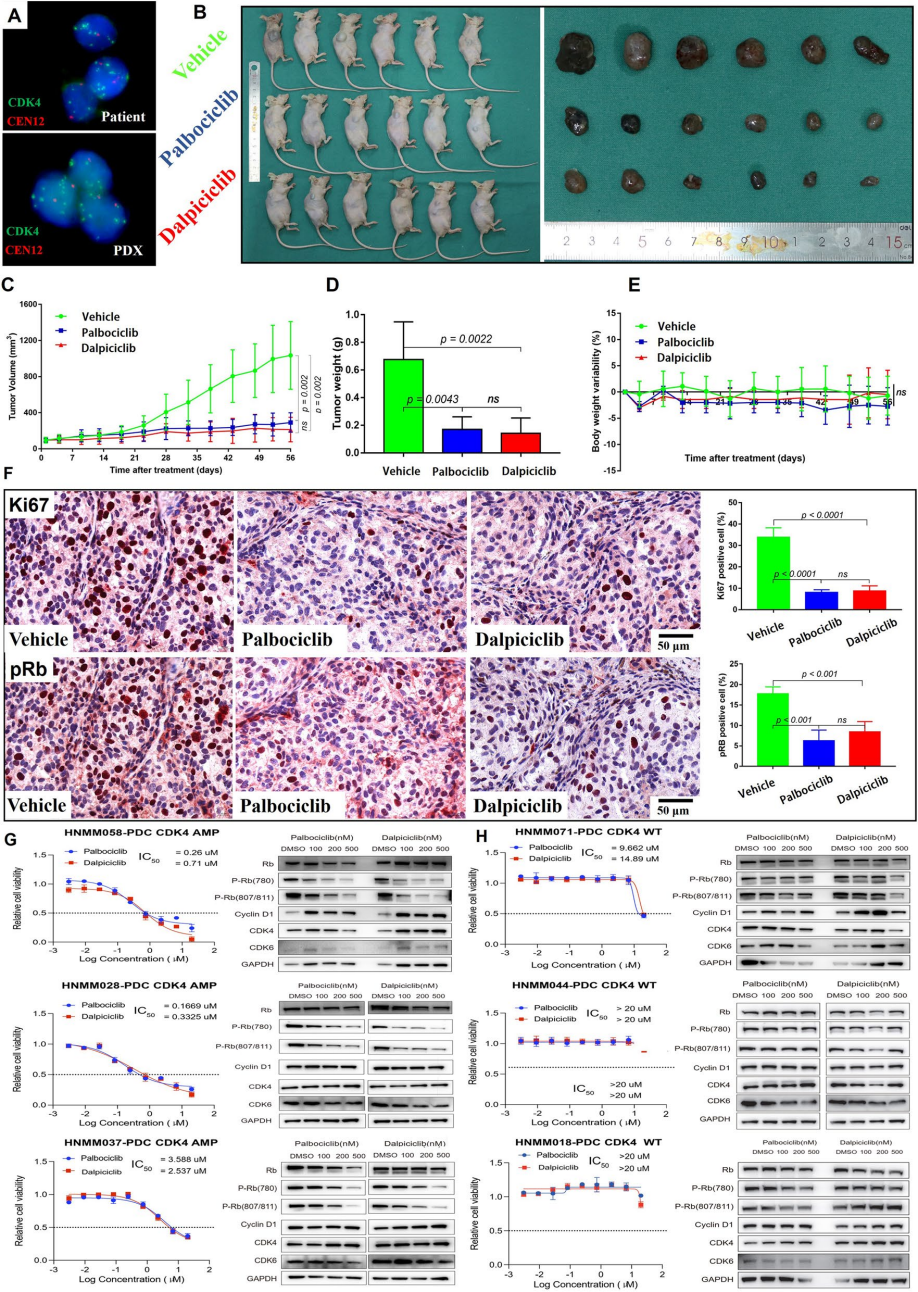
In vivo and intro validations of the palbociclib and dalpiciclib in HNMM PDX and PDC models. **A**–**C** Therapeutic efficacy of palbociclib and dalpiciclib in CDK4-amplified MM028-PDX (*n* = 6). **D** The weight of the tumors from different groups. **E** Evaluation of treatment toxicity in PDX models. Body weight change of mice during the treatment with palbociclib and dalpiciclib. **F** Immunoblot analysis of Ki67 and pRB expression in tumors after the last administration with vehicle, palbociclib and dalpiciclib. **G**, **H** The sensitivity of palbociclib and dalpiciclib in HNMM-PDC with CDK4 amplification (AMP) and without CDK4-amplified (wild type, WT). CDK4/6-RB signaling pathway and downstream proteins were detected by western blotting in the indicated groups. Scoring of Ki-67 and PRB staining are summarized as the mean ± SEM. Tumor volume, tumor weight, body weight, Ki67 expression, and pRB expression were analyzed with Student’s *t* test. ns, not significant

To pave the way for the clinical investigation on dalpiciclib in HNMM, we first evaluated the in vivo and in vitro anti-tumor efficacy of dalpiciclib using the stably passaged HNMM-PDX and HNMM-PDC. Palbociclib (CDK4/6 inhibitor) as a reference drug was also assessed to validate the results, which showed a promising anti-tumor effect in HNMM-PDX harboring CDK4 amplification in our previous study [3]. Consistent with our previous results, dalpiciclib and palbociclib profoundly suppressed the growth of HNMM-PDX xenograft tumors with CDK4 amplification, whereas a relatively weak suppression of PDX with CDK4 wild type when compared with vehicle was shown (Fig. 1A–D and Additional file 1: Table S1). Drug toxicities of both CDK4/6 inhibitors were all well tolerated, with no appreciable loss in body weight (Fig. 1E). We then used Ki-67 and pRB staining to assess the proliferative status and the key downstream factor of CDK4/6 activity after the treatment of dalpiciclib and palbociclib. Dalpiciclib and palbociclib resulted in a remarkable reduction in tumor proliferation rate and phosphorylated Rb levels determined by Ki-67 and pRB immunostaining than control group (Fig. 1F). The same results were also found by in vitro experiments. Dalpiciclib and palbociclib all displayed a concentration-dependent inhibition of tumor growth in HNMM-PDC harboring CDK4 amplification, but there was no inhibitory effect on HNMM-PDC with CDK4 wild type cells (Fig. 1G,H). Western blot analysis revealed decreased Rb phosphorylation in the HNMM-PDC treated with palbociclib or dalpiciclib when compared with cells treated with vehicle control (Fig. 1G,H). Thus, the in vivo and in vitro studies using the HNMM-PDX and HNMM-PDC models further validated the anti-tumor effects of dalpiciclib and palbociclib. No significant difference was observed between both dalpiciclib and palbociclib, indicating the promising therapeutic effect of CDK4/6 inhibitors in HNMM with CDK4 amplification.

### Patient characteristics and treatments

Between July 30, 2020, and February 1, 2023, a total of 26 patients were screened. Seventeen patients with advanced HNMM were enrolled in this study, received at least one dose of dalpiciclib, and were evaluable for safety (Additional file 2: Fig. S1). Detailed patient characteristics are presented in Table 1. The median age was 56 years (ranged 29–73 years) with 8 (47.1%) male patients. One subject who did not complete the first cycle and voluntarily withdrew from the study was excluded from efficacy analysis. Eight patients (47.1%) had ≥ 3 metastases sites, and the median baseline sum of diameters of target lesion was 55.9 mm (range:14.5–118 mm). Two patients (11.8%) were BRAF mutation, two (11.8%) were C-KIT mutation, and one (5.9%) was NRAS mutation. All 17 patients were CDK4 amplification (Additional file 1: Table S2). All patients underwent surgical excision as the primary treatment and had received prior therapy, with a median of one prior systemic treatment (range 1–3). Eleven patients (64.7%) had received chemotherapy combined with anti-angiogenic agents, 6 patients (35.3%) had received PD-1 inhibitor monotherapy, and 6 patients (35.3%) had received combined anti-PD-1 and anti-angiogenic agents, including bevacizumab and anlotinib (Table 1 and Additional file 1: Table S2). At data cutoff, 1 patient remained on treatment.

**Table 1:** Baseline patient characteristics

Characteristic	No	%
Sex		
Male	8	47.1
Female	9	52.9
Age, years		
Median	56	
Range	29–73	
ECOG PS		
0	1	5.9
1	16	94.1
TNM stage (AJCC 7th)		
IVA	1	5.9
IVB	7	41.2
IVC	9	52.9
CDK4 status		
Amplification	17	100
Previous lines of treatment, *n* (%)		
1 prior line	11	64.7
2 prior line	4	23.5
3 prior line	2	17.6
Prior therapy		
Interferon	14	82.4
Chemotherapy + anti-angiogenic agents	11	64.7
Anti-PD-1 monotherapy	6	35.3
Anti-PD-1 + anti-angiogenic agents	6	35.3
Mutation status		
BRAF	2	11.8
C-KIT	2	11.8
NRAS	1	5.9
PD-L1 status*		
Positive	1	5.9
Negative	16	94.1
No. of sites of metastasis		
< 3	9	52.9
≥ 3	8	47.1
BSLD (mm)		
Median	55.9	
Range	14.5–118	

*Abbreviations*: *ECOG PS*, Eastern Cooperative Oncology Group performance status; *CDK4*, cyclin-dependent kinase 4; *BSLD*, baseline sum of longest diameter; *AJCC*, American Joint Committee on Cancer

^*^Positive defined as ≥ 1% of tumor cells expressing PD-L1 by SP142 IHC staining

### Efficacy

**Fig. 2:**
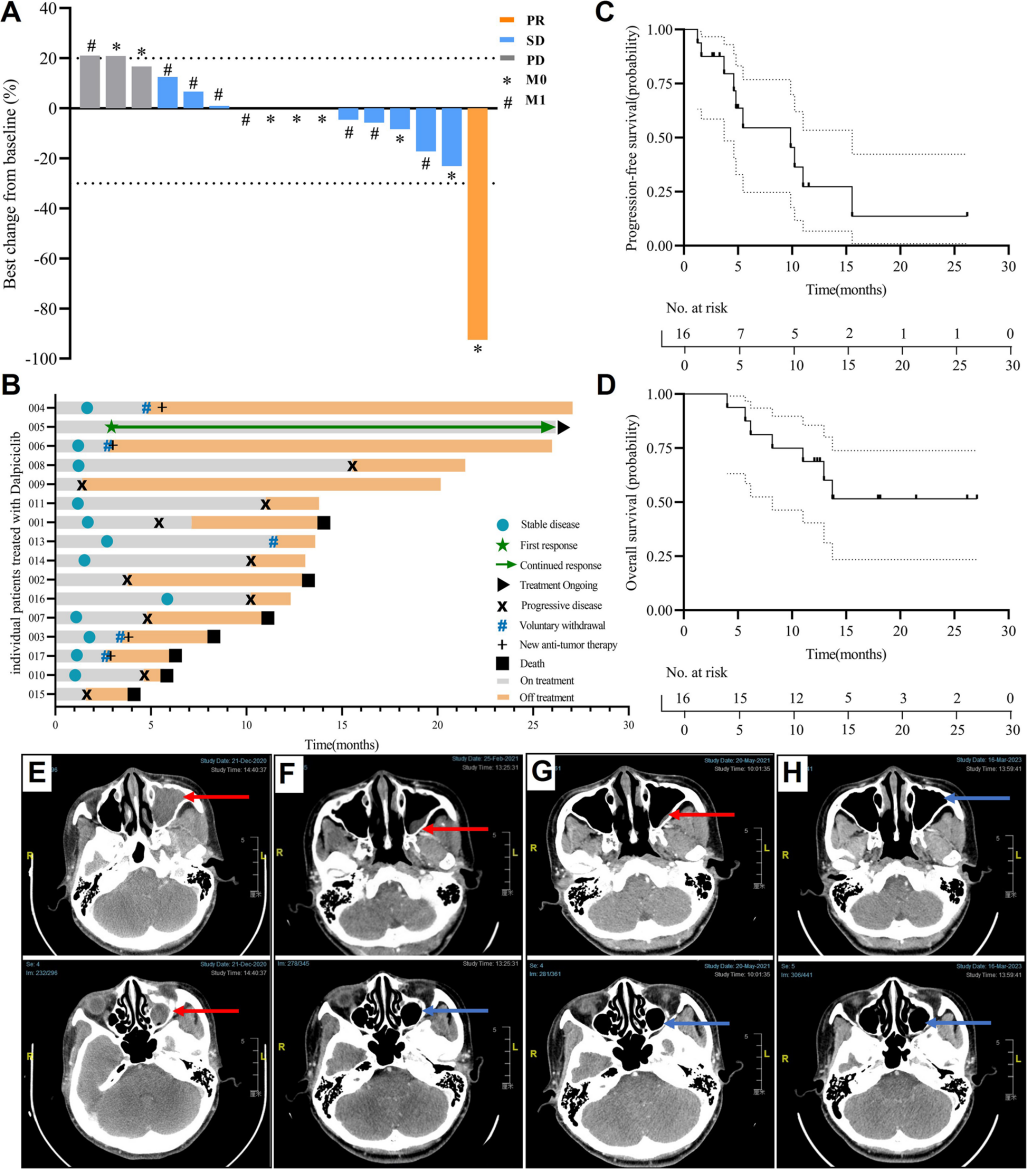
Clinical response and survival of patients with HNMM to dalpiciclib. **A** Maximum tumor percent change in target lesion from baseline in patients treated with dalpiciclib (*n* = 16). Individual patients are indicated by each bar, with * and # indicating the baseline M stage. **B** Swimming plot demonstrating the treatment exposure and duration of tumor response in the full analysis (FAS) population per RECIST v1.1 (*n* = 16). Time in study for all evaluable patients. Arrows indicate patients who remained in study at the data cutoff. Star indicates patient with partial response. Individual patients are indicated by each bar. **C** Kaplan–Meier curves of PFS, median PFS was 9.9 mo. (95% CI, 4.8-NA). **D** Kaplan–Meier curves of OS, median OS was NR. PFS, progression-free survival. OS, overall survival. RECIST, Response Evaluation Criteria in Solid Tumors. **E**, **F** The radiographic photographs of patient 005. The computed tomography scans at **E** baseline and after 2 months (**F**), 5 months (**G**), and 2 years (**H**) of treatment with dalpiciclib, demonstrating favorable tumor response (arrows) in 005 participant. CT on December 21, 2020, revealed the presence of left maxillary sinus solid occupation (red arrow). The lesion showed significant regression, and the outcome was a partial response of tumors after two cycles. There was an almost complete regression of tumor after treatment for five cycles (**G**), and there was no recurrence after two years following (blue arrow)

At data cutoff, 7 (43.8%) had died, 8 (50%) discontinued treatment, and 1(6.3%) patient remained on study among the 16. The median treatment duration was 4.6 months (range, 1.2 to 26.6 months). A decrease in target lesions of any size from baseline was observed in 6 patients (37.5%) (Fig. 2A). The confirmed ORR and DCR were 6.3% (1/16) and 81.3% (13/16), respectively (Table 2). Four patients with SD (#3, #4, #5, #17) dropped out before 24 weeks to receive new anticancer therapy, of whom 3 patients received PD-1 combined with vascular endothelial growth factor (VEGF) inhibition and 1 patient received paclitaxel combined with bevacizumab. Additionally, half of the patients (6/12) had a PR or SD for ≥ 24 weeks. The duration of study treatment for each patient is depicted in Fig. 2B. With a median follow-up of 12.8 months (range, 4.1 to 27.1 months), the estimated median PFS was 9.9 months (95% CI, 4.8-NA; Fig. 2C). The median OS was not reached; the estimated rate of OS at 12 months was 68.8% (95% CI, 0.494–0.957), and at 24 months, it was 51.6% (95% CI, 0.307–0.866) (Fig. 2D). One patient (6.3%) achieved a partial response according to RECIST v1.1 at 8 weeks, with a duration of response of 27 months (Fig. 2E,F and Additional file 2: Fig. S2). This patient remained on treatment, and the dalpiciclib dose was reduced to 100 mg for managing AEs. Three other patients had evidence of favorable response to treatment but did not meet RECIST v1.1, specifically, decreases in tumor size of at least 10%.

**Table 2:** Confirmed best overall response rates according to RECIST v1.1 (FAS)

	Dalpiciclib (*n* = 16)
Best overall response, *n* (%)	
PR	1 (6.3)
SD	12 (75.0)
PD	3 (18.7)
ORR (CR + PR), *n* (%)	1 (6.3)
(95% Cl)	(0.2–30.2)
Disease control rate (CR + PR + SD), *n* (%)	13 (81.3)
(95% Cl)	(54.4–96.0)

*Abbreviations*: *PD*, progressive disease; SD, stable disease; *CR*, complete response; *PR*, partial response

### Safety and tolerability

All patients experienced any grade of TRAEs, and most TRAEs were grades 1–2. Only 1 patient experienced grade 3 neutrophil count decreased and white blood cell count decreased, who was also the one with the best clinical outcome (PR). No grade 4 or 5 AEs were reported. The most common TRAEs were neutrophil count decreased (64.7%), white blood cell count decreased (64.7%) (Additional file 1: Table S3), fatigue (41.2%), and lymphocyte count decreased (29.4%). The drug was overall well tolerated, and no serious AEs or other significant AEs occurred. Two patients required dose reductions for toxicity: 1 because of hematologic toxicity and the other because of rash. No treatment-related death occurred during this study. No patient reported infection due to myelosuppression. No patient withdrew from therapy because of unacceptable toxicity (Table 3).

**Table 3:** Treatment-related adverse events

TRAEs	All grade, *n* (%)	Grade 3, *n* (%)
All TRAEs	17 (100.0)	1 (5.9)
White blood cell count decreased	11 (64.7)	1 (5.9)
Neutrophil count decreased	11 (64.7)	1 (5.9)
Fatigue	7 (41.2)	0
Lymphocyte count decreased	5 (29.4)	0
Rash	4 (23.5)	0
Anemia	2 (11.8)	0
Anorexia	2 (11.8)	0
Headache	2 (11.8)	0
Pruritus	1 (5.9)	0
Breast pain	1 (5.9)	0
Cough	1 (5.9)	0
Eczema	1 (5.9)	0
Skin hypopigmentation	1 (5.9)	0

### Baseline genetic features

**Fig. 3:**
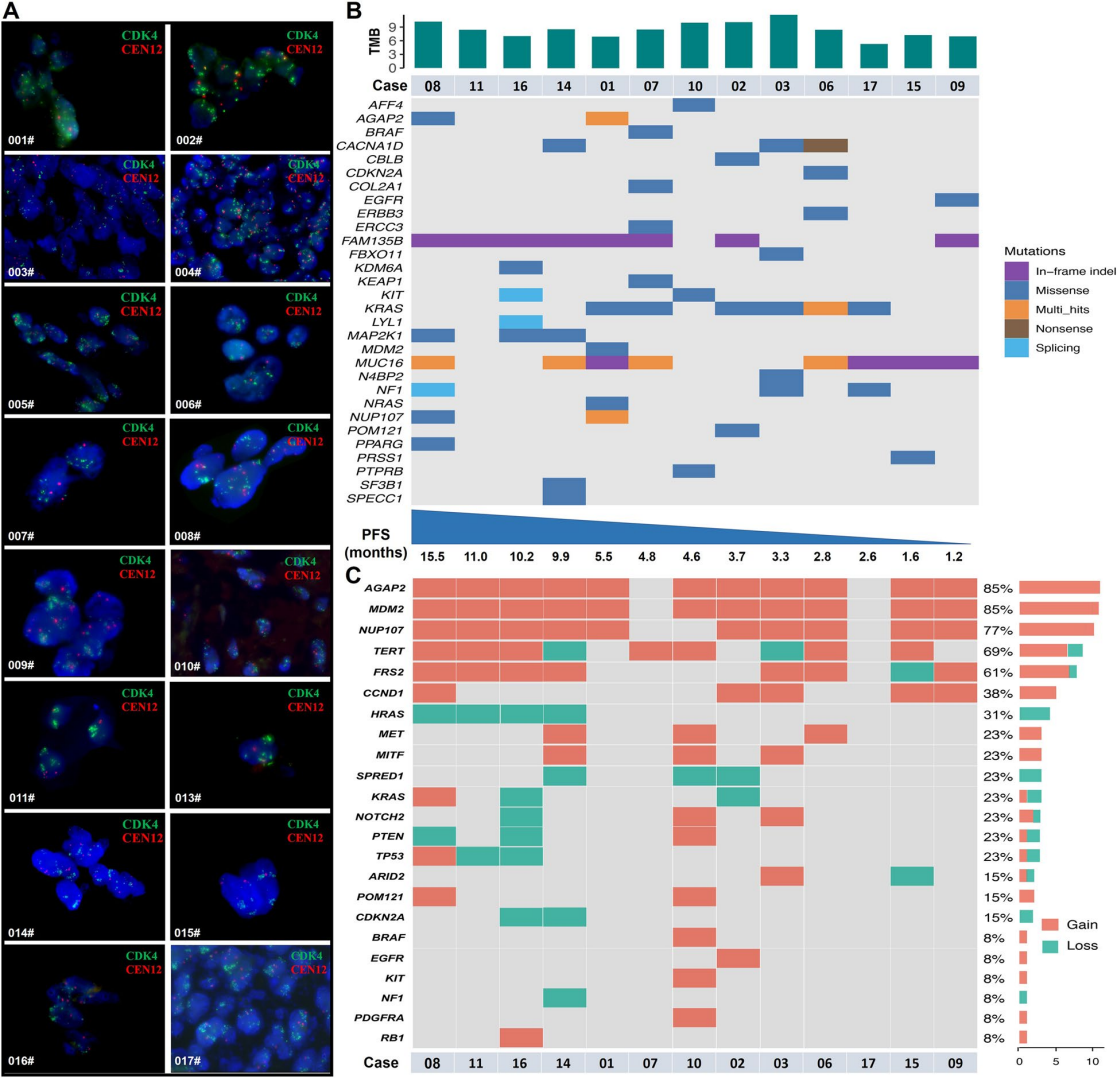
Baseline genetic feature of patients with HNMM received dalpiciclib. **A** Fluorescence in situ hybridization (FISH) results of patients included in evaluation. **B** Tumor mutational burden (TMB) in each tumor sample and spectrum of significant cancer genes identified by whole exome sequencing. **C** Somatic copy number alterations of known cancer genes

All 17 patients included in the study exhibited CDK4 amplification, with the results of FISH analysis for the 16 evaluated patients presented in Fig. 3A. High-coverage whole-exome sequencing was performed on baseline tumor samples of 13 patients. The mean TMB was 8.38 per Mb (range:5.28–11.69). The mutation landscape of each tumor is summarized in Fig. 3B. In total, 30 significant cancer genes were identified by MutSigCV analysis. All patients carried at least three mutated cancer genes, except for case 11 with only a FAM135B and case 15 with MUC16 and PRSS1 gene mutations. Notably, FAM135B was identified in 7 patients with PFS more than 4.8 months and in 2 patients with PFS less than 4.8 months. The somatic CNVs of known cancer genes are summarized in Fig. 3C. In our study, most of the patients were found to harbor AGAP2, MDM2, and NUP107 amplifications simultaneously, which was roughly the same as in our previous studies [3]. HNMM patients with CDK4 amplification were usually accompanied by these three gene amplifications. Interestingly, loss of HRAS was detected in four patients with PFS more than 9 months, whereas those without HRAS deletion was all with PFS less than 6 months.

## Discussion

MM is one of the most serious challenges for new melanoma therapies [21], possibly because of their rarity and its high rate of recurrence and metastasis as well as a lack of well-established therapeutic guideline. Normally, patients with mucosal melanomas are treated with the same regimens recommended for cutaneous melanoma despite data already available showing that they may be less effective. Novel therapeutic strategies are urgently needed to improve outcomes in patients with MM.

Targeting CDK4 as potential therapeutic opportunities in melanoma has been of interest for several years. Preclinical and clinical studies validate CDK4 as a potential target for therapeutic intervention in melanoma patients [1]. In Guo’s clinical trial, three patients had SD, and the best clinical response was also a PR in one (1/15) patient, who had CDK4 amplification and did not receive any previous systemic treatment [22]. Their results suggested palbociclib may be a promising therapeutic when applied as monotherapy for individual patients with melanoma. Previous studies conducted by our team [3], as well as other published data, have shown that aberrations of CDK4 and aberrant activation of the CDK4 signaling pathway are frequently found in MM [2, 12, 23–25]. HNMM is one of the major sites of MM, especially in Asian populations [26]. In this study, our preclinical results showed the antitumor effect of dalpiciclib in HNMM PDC and PDX models with CDK4 gain. Based on the results from our study, CDK4/6 inhibitor was hypothesized to be biologically effective in patients with HNMM harboring CDK4 amplification.

The current study met its primary endpoint. The disease control rate of 81% significantly exceeded the expected DCR rate of 40% for an active second line agent. The confirmed ORR and DCR were 6.3% and 81.3%, respectively. Despite low ORR, durable disease control lasting > 10 months was observed in 5 patients. One patient experienced a confirmed PR, who continues to receive dalpiciclib on a compassionate-use, with a duration of more than 27 months at the last assessment (as of March 30, 2023). With a median follow-up of 12.8 months, the estimated median PFS was 9.9 months. Although overall survival data are currently immature, the probability of 12-month survival reached 68.8% (95% CI, 0.494–0.957), and the 24-month OS rate was 51.6% (95% CI, 0.307–0.866). Our results showed that dalpiciclib was efficient and may bring durable benefit for patients with advanced HNMM harboring CDK4.

Among our participants, patient 005# showed significant amplification of the CDK4 gene (ratio greater than 10). At the same time, dalpiciclib also demonstrated good therapeutic effects on this patient. However, based on existing experimental data, the amplification times of CDK4 does not seem to be directly proportional to the therapeutic effect. This may be related to the small sample size, and further studies with a larger number of samples and extended follow-up are needed. In addition to testing dalpiciclib in HNMM patients characterized by CDK4 amplification, this study specifically performed whole-exome sequencing for enrolled patients. And our results indicated that CDK4/6 inhibitor might have a better efficacy on MM with specific gene mutations. For instance, patients harboring FAM135B mutation and loss of the HRAS and CDKN2A might have a better PFS. Considering the tumor burden with an average diameter of about 56 mm was relatively high, the therapeutic effect was satisfactory. Patients with relatively small tumor target lesion diameter may have a relatively promising duration of disease control at the time of treatment (Additional file 1: Table S4 and Additional file 2: Fig. S3), which may indicate that single agent dalpiciclib may be more therapeutically beneficial for HNMM patients with lower tumor burden.

With respect to safety, the toxicity profile of dalpiciclib in MM was tolerable. Most of the AEs were of grade I or II. There were no treatment-related serious AEs reported, and all AEs resolved without sequelae. The most commonly reported TRAEs in this study included neutropenia/leukopenia, fatigue, nausea, diarrhea, or alopecia, which was consistent with previous studies of dalpiciclib in breast cancer and the other CDK4/6 inhibitor, palbociclib, in melanoma [14, 22]. Of note, one patient had skin hypopigmentation on face, similar to vitiligo during treatment. CDK4/6 inhibitor-induced vitiligo-like lesions is a rare and unpredictable adverse event. Currently, vitiligo is considered as a sign of good prognosis for melanoma patients with immunotherapy [27]. The patient had stable disease for approximately 9 months. The association of depigmentation as the side effect of CDK 4/6 inhibitor with prognosis warrants further attention.

Despite the satisfactory results, it is important to acknowledge that there are limitations in this study. Due to the infrequency of cases involving HNMM with CDK4 amplification, the study was structured as an uncontrolled, single-arm, open-label trial. One significant limitation of this approach is the lack of a control group for comparative analysis. Given the exploratory nature of this pilot study, the primary objective was not to establish the efficacy of dalpiciclib in treating patients with advanced HNMM harboring CDK4 amplification but rather to assess feasibility and identify optimal methodologies for future trials. Despite the limited response rate, likely impacted by the small sample size, our results suggest that dalpiciclib could delay disease progression in patients with advanced head and neck mucosal melanoma harboring CDK4 amplification. Further research is needed to establish the effectiveness of dalpiciclib and other CDK4 inhibitors, which may have widespread utility in melanoma at various anatomical locations.

To our knowledge, this is the first phase II clinical trial to date to show the promising antitumor activity and safety profile of CDK4/6 inhibitor in patients with advanced HNMM harboring CDK4 amplification. Mucosal melanomas are complex tumors with multiple chromosomal abnormalities, so precise treatment of patients with genetic profile is particular important. The current study demonstrates that treatment with a selective CDK4/6 inhibitor leads to favorable PFS for HNMM patients harboring CDK4 amplification.

## Conclusions

In conclusion, our preliminary results suggests that dalpiciclib may be a promising therapeutic strategy for patients with MM harboring CDK4 amplification and/or mutations in CDK4 pathway when applied as monotherapy or in combination with other therapeutic approaches.

### Supplementary Information

Additional file 1: Tables S1-4. Table S1. Inhibitory effects of palbciclib and dalpiciclib on tumor growth in HNMM-PDX models. Table S2. Baseline clinical characteristics and previous systemic therapies. Table S3. Baseline and lowest level of neutrophil and white blood cell counts for each patient. Table S4. Tumor burden and patient outcomes.

Additional file 2: Fig. S1. Diagram showing flow of patients.

Additional file 3: Fig. S2. The clinical photographs of patient 005. The intraoral view at (A) baseline and after 2 months (B) and 2 years (C) of treatment with dalpiciclib, demonstrating favorable tumor response in 005 participant.

Additional file 4: Fig. S3. The relationship between diameter of target lesions and progression-free survival.

## Data Availability

The data generated in this study are not publicly available due to information that could compromise patient privacy but are available upon reasonable request from the corresponding author.

## Abbreviations

AEs: Adverse events
CDK 4: Cyclin-dependent kinase 4
CI: Confidence intervals
CNVs: Copy number variations
CTCAE: Common terminology criteria for adverse events
DCR: Disease control rate
ECOG: Eastern oncology cooperative group
FAS: Full analysis set
FFPE: Formalin-fixed paraffin embedded
FISH: Fluorescence in situ hybridization
HER2: Human epidermal growth factor receptor 2
HNMM: Head and neck mucosal melanoma
IACUC: Institutional animal care and use committee
ICI: Immune checkpoint inhibitor
IHC: Immunohistochemistry
MM: Mucosal melanoma
ORR: Objective response rate
OS: Overall survival
PDC: Patient-derived tumor cell
PDX: Patient-derived xenografts
PFS: Progression-free survival
PR: Partial response
RECIST: Response evaluation criteria in solid tumors
SAS: Safety analysis set
SD: Stable disease
TGI: Tumor growth inhibition
TMD: Tumor mutation burden
TRAEs: Treatment-related adverse events
VEGF: Vascular endothelial growth factor
WGS: Whole genome sequencing
