# Supplementary figures and images for: The efficacy and safety of dalpiciclib, a cyclin-dependent kinase 4/6 inhibitor, in patients with advanced head and neck mucosal melanoma harboring CDK4 amplification
Source: BMC Med. 2024 May 29;22:215. doi: 10.1186/s12916-024-03431-x (PMC11134887; doi:10.1186/s12916-024-03431-x)

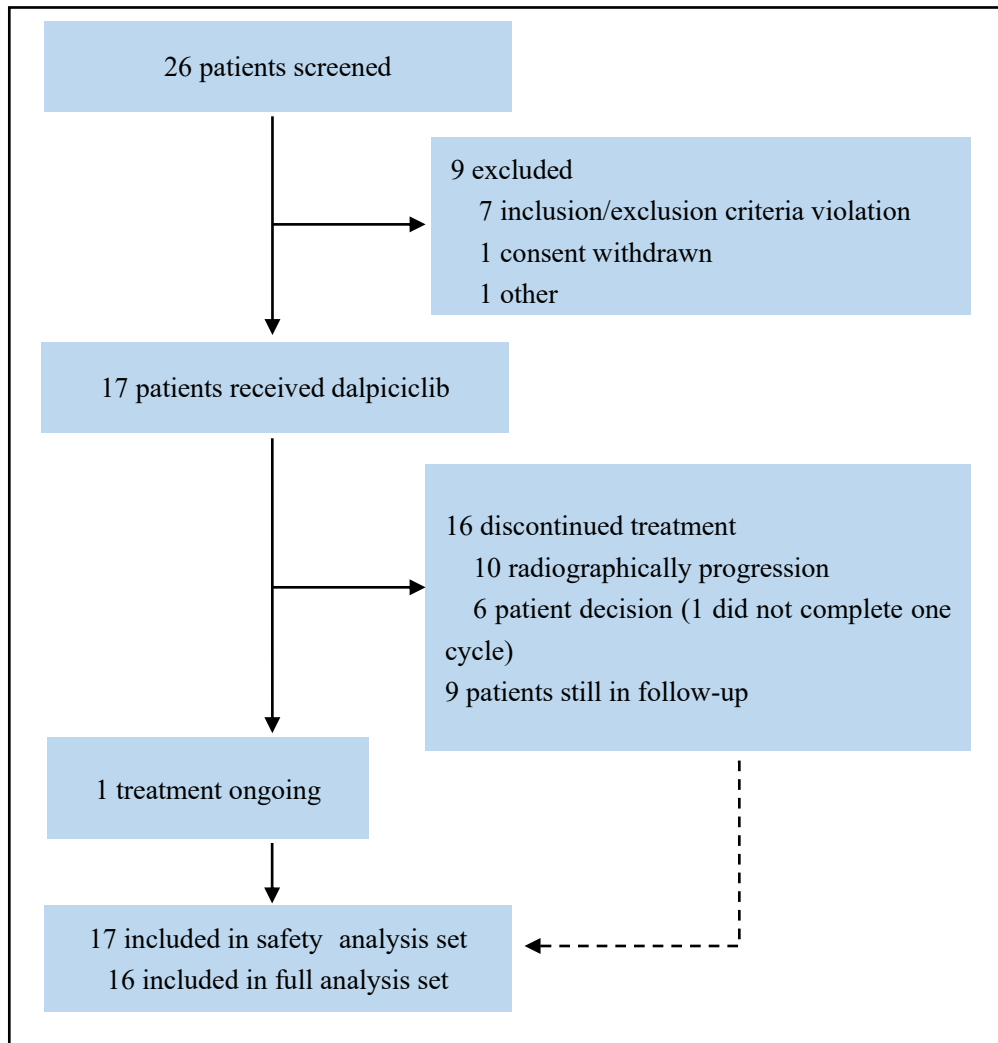

**Fig S1.** Diagram showing flow of patients.

Supplement: Supplementary file 2 — Additional file 2: Fig. S1. Diagram showing flow of patients. [file 12916_2024_3431_MOESM2_ESM.pdf]

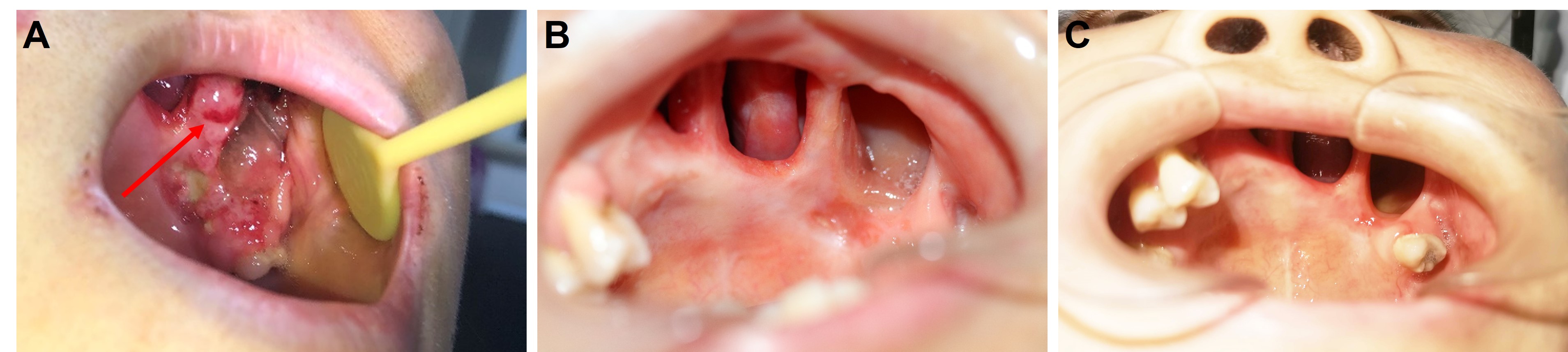

Supplement: Supplementary file 3 — Additional file 3: Fig. S2. The clinical photographs of patient 005. The intraoral view at (A) baseline and after 2 months (B) and 2 years (C) of treatment with dalpiciclib, demonstrating favorable tumor response in 005 participant. [file 12916_2024_3431_MOESM3_ESM.jpg]

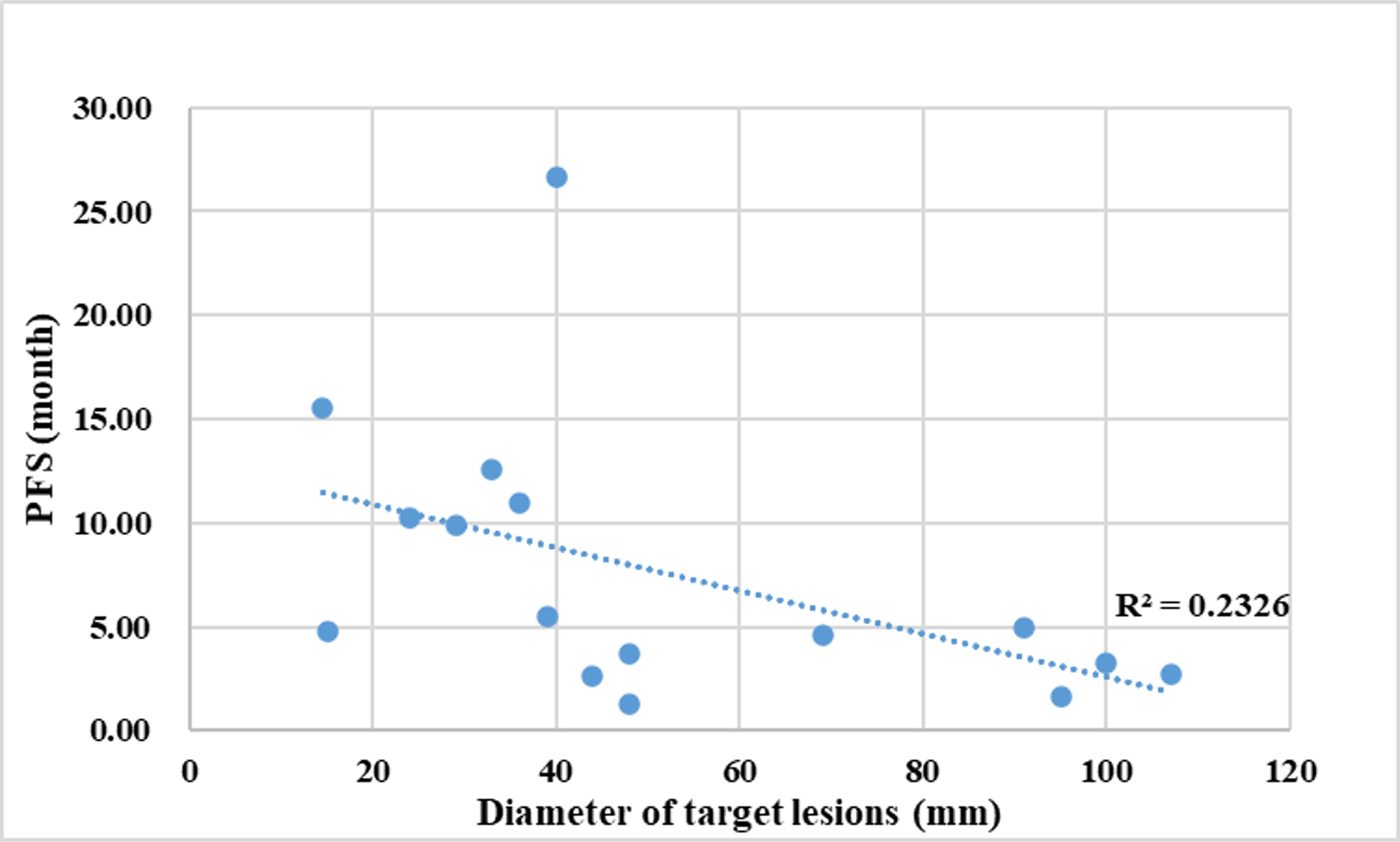

Supplement: Supplementary file 4 — Additional file 4: Fig. S3. The relationship between diameter of target lesions and progression-free survival. [file 12916_2024_3431_MOESM4_ESM.jpg]
